# Illicit opioids, methamphetamine/amphetamine, and cocaine use are associated with loss of housing and delay with becoming rehoused across seven diverse cohorts with and without HIV in the United States and Canada

**DOI:** 10.1016/j.dadr.2026.100483

**Published:** 2026-10-05

**Authors:** Joseph A. Delaney, Lydia N. Drumright, Michael E. Newcomb, M.-J. Milloy, Madison Shea Smith, Raul Mandler, Brenton Swenson, Carolyn A. Fahey, Kora DeBeck, Shruti Metha, Becky L. Genberg, Brian Mustanski, Bridget M. Whitney, Ryan P. Kyle, Patrick Janulis, Michele Kipke, Marjan Javanbakht, Pamina M. Gorbach, Gregory Kirk, Heidi M. Crane, Kanna Hayashi, Geetanjali Chander

**Affiliations:** a Department of Medicine, University of Washington, Seattle, WA, USA; b Feinberg School of Medicine, Northwestern University, Chicago, IL, USA; c Department of Medicine, University of British Columbia, Vancouver, BC, Canada; d Division of Therapeutics and Medical Consequences, National Institute on Drug Abuse, National Institutes of Health, Bethesda, MD 20892, USA; e Faculty of Health Sciences, Simon Fraser University, Vancouver, BC, Canada; f Department of Medicine, Johns Hopkins University, Baltimore, MS, USA; g Keck School of Medicine, University of Southern California, CA, USA; h Fielding School of Public Health, University of California Los Angeles, CA, USA; i British Columbia Centre on Substance Use, Vancouver, BC, Canada

## Abstract

**Background:**

There is a need for evidence as to whether substance use (by type) has a different relation with (1) loss of housing and (2) regaining housing among people who use substances.

**Methods:**

Seven cohorts, all part of the HIV SUCCESS consortium, contributed data between 2005 and 2024 to estimate time to change in housing status in longitudinal models with six-month intervals. To examine associations between substance use and loss of housing, we first included all participants at their first housed visit and estimated time to housing loss. To examine associations with rehousing, we included participants who were unhoused at any point prior to their final study visit and estimated time to regain housing. Housing status and substance use were self-reported. For all analyses we used adjusted time-updated Cox models with a separate model for each substance. Cohort-specific results were then pooled using a random effects meta-analysis approach with inverse variance weights.

**Results:**

Among 4931 housed participants followed for an average of 4.2 years, current use of methamphetamine/amphetamines (hazard ratio [HR]=1.81, 95% confidence interval [CI]:1.47, 2.23), opioids (HR=1.68, 95%CI:1.39, 2.02) and cocaine (HR=1.39, 95%CI:1.14, 1.69), were associated with a significantly faster rate of becoming unhoused, once adjusted for age, sex, race/ethnicity and year of study enrollment. Among 1901 unhoused participants, followed for an average of 1.4 years, methamphetamine/amphetamines (HR=0.80, 95%CI:0.70, 0.91) opioids (HR=0.85, 95%CI:0.76, 0.95) and cocaine (HR=0.83, 95%CI:0.75, 0.92) were associated with regaining housing at a slower rate.

**Conclusions:**

Opioid, methamphetamine/amphetamine, and cocaine use are all associated with adverse housing outcomes.

## Highlights

• The type of substance being used was important in predicting housing outcomes.
• Cocaine/crack use had worse housing outcomes among cohorts with older participants.
• The use of methamphetamines is associated with the poorest housing outcomes.
• Frequent use of methamphetamines was a especially strong predictor of poor outcomes.

## 1 Introduction

Rates of substance use are known to be elevated among people who are unhoused,(Stringfellow et al., 2016, Doran et al., 2018, Richards and Kuhn, 2023, Assaf et al., 2025) creating an additional risk for health harms in this already vulnerable, stigmatized, and underserved population. As the unhoused population has been increasing in recent years, it becomes ever more important to understand the drivers of becoming and staying unhoused (Dombrowski et al., 2025, Tsai and Lampros, 2025). However, the association between substance use and changes in housing status is complex and multifactorial (Malfitano and Prado, 2025). It is not always clear what role substance use has played in housing outcomes versus housing outcomes creating the conditions for active substance use (Chassman et al., 2022). Some literature suggests that non-alcohol substance use may play a limited role in determining the transition from being housed to unhoused (McVicar et al., 2015) and so it is key to determine if substance use prior to becoming unhoused is related to the subsequent loss of housing (Austin et al., 2021), as opposed to being a consequence of this change in housing status. One important consideration that requires further elucidation is whether the type of substance being used is an important element of the strength of this known association.

In particular, the use of illicit opioids, methamphetamine/amphetamine, and cocaine often leads to adverse health outcomes (including increased mortality as well as health issues such as overdose, depressive symptoms, and reduced ability to regularly take medications) among people who use drugs, as compared to people who do not use drugs, and are therefore of special interest (Delaney et al., 2018, Zolopa et al., 2022, Ma et al., 2024, Shearer et al., 2024). Numerous approaches have been considered to support people who use drugs who experience housing instability, including evidence-based practices such as Housing First that emphasize providing immediate access to permanent housing without prerequisites such as sobriety (O’Campo et al., 2022, Isaak et al., 2024). Estimating the magnitude of the association between current substance use and time to being unhoused is key to understanding these populations.

While housing is an important issue for all people who use drugs, there are also important subpopulations, including people with HIV. In particular, it is important to better understand whether the challenges of living with HIV confounds the role of current substance use as a barrier to improving housing outcomes, especially given the known increase in morbidity due to HIV infection (Milloy et al., 2012) and increased health care utilization (Milaney et al., 2021) among those individuals. It is also possible that this subpopulation might even have improved health outcomes due to access to better resources such as healthcare programs targeted at treatment for people with HIV.

We conducted a study using seven cohorts in the United States and Canada to characterize the longitudinal association between opioid, amphetamine, and cocaine use with both (1) time to loss of housing and (2) time to regaining housing, as estimates of the rate of housing transitions for participants with different substance use exposures. The study populations from the consortium were pooled with the intention to be broad in both entry requirements and geography, with the goal of creating an estimate that can be broadly applied, while still retaining the ability to detect important levels of heterogeneity between the cohorts. We aimed to examine whether analysis by specific substance use further elucidates associations in this population.

## 2 Methods

We included seven cohorts in this study, to allow for both more generalizable conclusions and to enhance power for greater precision in the estimates of the association between substance use and housing outcomes. All of these cohorts are funded by the United States’ National Institute on Drug Abuse (NIDA) with the goal of better understanding the impact of substance use on the HIV epidemic in the US and Canada via longitudinal data collection. The AIDS Linked to the IntraVenous Experience (ALIVE) Study is a prospective community-based cohort study in Baltimore, MD, which was designed to characterize the incidence and natural history of HIV infection among people who inject drugs (Vlahov et al., 1991). Follow-up visits were every six months in ALIVE. The RADAR cohort is a longitudinal dyadic-network cohort of 1200 diverse, young men who have sex with men (MSM) based in Chicago, IL intended to better understand the interplay between HIV and substance use in this population (Janulis et al., 2018). Follow-up visits were every six months in RADAR. The Healthy Young Men (HYM)/TRUTH cohort study is a longitudinal research study tracking young people’s health behaviors, substance use, and psychosocial status to characterize risk factors for adverse outcomes during the transition from adolescence to adulthood (Azucar et al., 2022). Follow-up visits were every six months in HYM/TRUTH. The MSM and Substances Cohort at UCLA Linking Infections, Noting Effects (mSTUDY) is a cohort study in among MSM of color focused on examining the role of substance use on HIV outcomes (Javanbakht et al., 2019). Follow-up visits were every six months in mSTUDY. Finally, there were three pre-harmonized open prospective cohort studies of community-recruited people who use drugs in Vancouver, including the Vancouver Injection Drug Users Study (VIDUS; enrolling adults [aging 18 or older] at risk of HIV who inject drugs), the AIDS Care Cohort to Evaluate exposure to Survival Services (ACCESS; enrolling adults living with HIV who use unregulated drugs) and the At-Risk Youth Study (ARYS; enrolling street-involved youth aged 16–25 years who use unregulated drugs) (Strathdee et al., 1997, Strathdee, 1998, Wood et al., 2006). Participants follow-up visits in ACCESS, ARYS, and VIDUS are semi-annual, giving the same follow-up interval as the four US cohorts and the similar location and survey design allowed for the direct pooling of these three cohorts for analysis.

Pooling cohorts is a well-understood approach to answering important questions that no single cohort could answer, and this approach has been successful in past substance use consortiums (Chandler et al., 2017, Gorbach et al., 2021, Jenkins et al., 2022). Data used in this study came from cohort data collection periods encompassing 2005–2024, varying by cohort. ALIVE data were collected between 2010 and 2024, RADAR data were collected between 2015 and 2024, HYM/Truth data were collected between 2013 and 2024, mSTUDY data were collected between 2014 and 2023, whereas ACCESS, ARYS, and VIDUS data were collected between 2005 and 2023. These cohorts also include people living with HIV (PWH), who are already at higher risk of substance use as a syndemic (Ma et al., 2024) and form an especially vulnerable sub-population among people who use drugs.

Ethics approval for use of pre-collected deidentified cohort data was given by University of Washington Human Subjects Division (HSD) under STUDY00016838. Indigenous participants in Vancouver were not included in the cohort consortium dataset due to local ethics rules around data sharing, intended to ensure that studies that include these participants align with Indigenous ways of knowing and self-determination. Individual cohorts had their studies approved by local institutional review boards and all participants completed informed consent.

### 2.1 Exposure

We categorized self-reported substance use based on participant surveys into a “currently exposed” variable for the three main substances of interest: Illicit opioid (not including prescription opioids), methamphetamine/amphetamine, and cocaine, hereafter called opioids, amphetamines, and cocaine for simplicity. Cocaine includes all forms of cocaine including both powder cocaine and crack cocaine. Amphetamines could not be divided into methamphetamine versus other forms as not all cohorts subdivided the amphetamine category. We focused on any current use (defined as reporting any use in the past six months on the date of the interview) as the most relevant measure of drug exposure and all cohorts measured use in the prior six months for these substances (Gorbach et al., 2021). Using cocaine as an example, in ALIVE the question was “*These next questions are about the specific drugs that you may have used in the last six months. Remember that all of your responses are completely confidential*” and had responses for *“Inject cocaine by itself?”*, *“Smoke crack, ready rock, freebase, or “hard” cocaine?”,* and *“Sniff or snort cocaine?”* as well as questions on drug combinations (e.g., speedball use) that were aggregated to create the current use cocaine variable. In RADAR the question was of the form “*In the PAST 6 MONTHS, how often have you used cocaine/crack?”*. Frequency of current use, specifically parameterized as either infrequent use (<weekly) and frequent use (at least weekly), of specific substances were examined in sensitivity analyses to see if there was a dose-response with higher frequencies of use. HYM/TRUTH, mSTUDY, ACCESS, ARYS, and VIDUS also asked about injected and non-injected cocaine (and the other substances of interest) in the past six months.

### 2.2 Outcome

The outcome was self-reported housing status, as collected both at cohort baseline (operationally defined as the earliest visit in the time window of interest that the participant was eligible to be included) and during follow-up visits. Being unhoused was, for all seven cohorts, defined as self-report of being unhoused during the past 6 months at a study interview. Changes in the outcome were determined based on self-report of being housed/unhoused at subsequent 6-month visits for each cohort (and having it be discordant with a prior housing self-report). For example, RADAR asked the question *“Which of the following best describes your current living situation?”* and considered the participant unhoused if they reported *“No permanent address (for example: you are homeless, squatting, etc.)”*. Not all cohorts distinguished between stable and unstable housing (Fahey et al., 2025) so our contrast was whether or not the participant reported that they were currently housed or unhoused. In the very infrequent cases where housing status was missing for a study participant in one of the seven cohorts, the last reported housing status was carried forward. The date of the change in housing status (which is the declared study endpoint) was taken as the date that discordant housing data from baseline was first reported.

### 2.3 Analysis: time to becoming unhoused

We first examined associations of current substance use with time to loss of housing. A participant’s first visit where they reported being housed was defined as baseline for this analysis and were included in the study as long as they also had at least one subsequent visit. Participants follow-up ended at the first visit that they reported being unhoused (i.e., had the event), when they were lost to follow-up (did not return for any follow-up visits), or at their final visit if they never reported being unhoused. Covariates adjusted for were age at first housed visit, sex, race/ethnicity – as reported by interview responses -- and year of study enrollment, as reported by the cohort. We also adjusted statistical models for baseline depressive symptoms as measured by a psychometric score as a sensitivity analysis, to see if mental health might play a confounding role in these associations. Because the cohorts used different instruments to assess depressive symptoms, the PROMIS depression scale, the Center for Epidemiologic Studies Depression Scale (CES-D), or Brief Symptom Inventory (BSI), we used a cut-off of > 58.6 for PROMIS and > 20 for CES-D based on prior literature (Choi et al., 2014, Kim et al., 2017) to indicate the presence of depressive symptoms. BSI depression symptom data were converted to the PROMIS T-score as previously described (Tang et al., 2022). As a second sensitivity analysis we examined whether adjustment of estimates for HIV infection influenced our estimates of association since PWH have more complex health care needs and it was unclear whether this might influence associations. Overall, 879 of 61,237 (1.4%) total missing housing status entries were replaced by carrying forward the prior value across the full dataset, which we considered to be a negligible amount of missing data, justifying last value carried forward approaches to missing data (this was for just the housing variable, other variables also had some missing data but this approach was not used for them) (Engels, 2003, Enders, 2025). We also considered all three substances (MCO) pooled as a sensitivity analysis to provides estimates of the association that were comparable to prior research.

### 2.4 Analysis: time to regain housing

To examine associations with rehousing, we examined participants who were unhoused at any point prior to their final study visit. Participants entered analysis at their first visit where they reported being unhoused, and that visit was not the participant’s final visit on record. We did not allow participants who cycled back and forth between being housed and unhoused to enter the cohort multiple times. While this is a realistic scenario, we wanted to estimate the association of substance use with the next change in state (to estimate the maximum credible effect of a substance use intervention on the next housing status change) and so did not consider repeated housed/unhoused cycles for participants. We used the same adjustment strategy and sensitivity analysis strategy as in the prior analysis. Participants were followed until their first subsequent visit where they reported regaining housing, at the time of loss to follow-up, or at the final visit in dataset if the participant did not report regaining housing. We also considered all three substances (MCO) pooled as a sensitivity analysis.

### 2.5 Statistical models

The cohorts, ALIVE, HYM/TRUTH, mSTUDY, and RADAR were analyzed separately, and then the similarly designed ACCESS, ARYS, and VIDUS were pooled and then analyzed. We used a Cox proportional hazards model adjusted for sex, race/ethnicity, age, year of first study visit, and current substance use. Each model included a single substance (i.e., amphetamines, cocaine, opioids) due to collinearity concerns when putting all of the substances in a single statistical model. Current substance use was a time-updated variable. In particular, substance use and housing status were both updated at each six-month study visit, based on participant self-report and would typically be answered simultaneously. Participants were censored when they had an event, when they were lost to follow-up, or at the end of the cohort period. We were not able to distinguish reason for loss to follow-up and some cohorts likely had participants where death was the reason for censoring due to loss to follow-up given prior reports on mortality in these cohorts (Sun et al., 2022). Estimates were combined using pooling methods developed for meta-analyses, and we used a random effect approach with inverse variance weights for all analyses. Friedenreich (1993) heterogeneity was screened for with an I^2^ statistics, with additional exploration if this showed evidence of important heterogeneity between estimates (Lin, 2020). The ALIVE cohort had to be excluded from frequency analyses done as part of the sensitivity work due to only recent collection of frequency data. All analyses were performed in Stata version 19.0, although preliminary analyses were initially conducted in R version 4.3.2 with the packages 'survival' for the Cox model and 'meta' for the pooled analysis.

## 3 Results

Across the seven cohorts, the first group of participants (housed) included 4931 participants who were followed for an average of 4.2 years (5.8 years among those who retained housing over all of follow-up and 3.1 years among those who eventually lost housing) and the second group of participants (unhoused) included 1901 unhoused participants who were followed for a mean of 1.4 years before the end of study follow-up (1.3 years among those who were rehoused and 2.0 years among those who were not). Among the housed participants, 31% lost housing during follow-up. Among the unhoused participants, 1062 of 1901 participants (56%) had transitioned back to housing by the time of their next study visit and 90% regained housing over the full follow-up. Mean time between visits was 224 days (Q1-Q3:180–208 days).

The housed participants were mostly male (80%) with a mean age of 35 years (standard deviation [SD]: 14) at baseline (Table 1). The unhoused participants were also mostly male (78%) and slightly older at a mean age of 38 years (SD: 13), although cohorts varied in mean age (Table 2). HIV status was almost the same (24% versus 29%) between the housed and unhoused populations but the proportion of participants showing depressive symptoms was lower among the housed (33%) than the unhoused (52%). The RADAR and HYM/TRUTH cohorts had notably proportions of participants reporting depressive symptoms at baseline than the proportion reported by the participants from the other cohorts.

**Table 1:** Demographic, health and substance use characteristics among participants by cohort and overall at the first housed visit.

	**ALIVE** **(N = 1751)** **N (%)** **Mean (+SD)**	**HYM/TRUTH** **(N = 738)** **N (%)** **Mean (+SD)**	**RADAR** **(N = 1264)** **N (%)** **Mean (+SD)**	**ACCESS/ARYS/VIDUS** **(N = 715)** **N (%)** **Mean (+SD)**	**mSTUDY** **(N = 463)** **N (%)** **Mean (+SD)**	**Overall** **(N = 4931)** **N (%)** **Mean (+SD)**
**Age**	49.9 (+ 8.6)	22.7 (+ 2.5)	21.4 (+ 3.1)	36.1 (+ 11.1)	32.0 (+ 6.9)	34.8 (+ 14.2)
**Male vs Female**	1147 (65.5)	576 (78.1)	1264 (100)	513 (71.8)	463 (100)	3963 (80.4)
**Race/Ethnicity**						
White	305 (17.4)	25 (3.4)	337 (26.7)	628 (87.8)	34 (7.3)	1329 (27.0)
Black/ African American	1400 (80.0)	188 (25.5)	403 (31.9)	24 (3.4)	184 (39.7)	2199 (44.6)
Latine/Hispanic	4 (0.2)	431 (58.4)	385 (30.5)	0	230 (49.7)	1050 (21.3)
Another Race/Ethnicity	42 (2.4)	94 (12.7)	139 (11.0)	63 (8.8)	15 (3.2)	353 (7.2)
**Current Meth/Amphetamine Use**	41 (2.3)	92 (12.5)	109 (8.6)	286 (40.0)	178 (38.4)	706 (14.3)
**Current Cocaine Use**	747 (42.7)	116 (15.7)	143 (11.3)	471 (65.9)	100 (21.6)	1577 (32.0)
**Current Opioid Use**	758 (43.3)	34 (4.6)	56 (4.4)	439 (61.4)	20 (4.3)	1307 (26.5)
**Diagnosed with HIV**	540 (30.8)	59 (8.0)	188 (14.9)	164 (22.9)	252 (54.4)	1203 (24.4)
**Depressive Symptoms (≥ Moderate)**	593 (33.9)	190 (25.8)	345 (27.3)	283 (39.6)	233 (50.3)	1644 (33.3)
**Lost Housing at Any Follow-Up**	588 (33.6)	111 (15.0)	134 (10.6)	470 (65.7)	223 (48.2)	1526 (31.0)
**Years of Follow-Up**	5.5 (+ 4.6)	3.6 (+ 2.5)	5.1 (+ 3.0)	6.1 (+ 5.7)	3.3 (+ 2.5)	5.0 (+ 4.1)

**Table 2:** Demographic, health and substance use characteristics among participants by cohort and overall at the first unhoused visit.

	**ALIVE** **(N = 779)** **N (%)** **Mean (+SD)**	**HYM/TRUTH** **(N = 113)** **N (%)** **Mean (+SD)**	**RADAR** **(N = 125)** **N (%)** **Mean (+SD)**	**ACCESS/ARYS/VIDUS** **(N = 591)** **N (%)** **Mean (+SD)**	**mSTUDY** **(N = 293)** **N (%)** **Mean (+SD)**	**Overall** **(N = 1901)** **N (%)** **Mean (+SD)**
**Age**	48.4 (+ 9.9)	24.2 (+ 2.3)	25.5 (+ 3.5)	35.2 (+ 12.2)	32.6 (+ 6.9)	38.9 (+ 12.9)
**Male vs Female**	541 (69.5)	100 (88.5)	125 (100)	425 (71.9)	293 (100)	1484 (78.1)
**Race/Ethnicity**						
White	214 (27.5)	3 (2.7)	13 (10.4)	522 (88.3)	36 (12.3)	788 (41.5)
Black/ African American	542 (69.6)	51 (45.1)	74 (59.2)	23 (3.9)	127 (43.3)	817 (43.0)
Latine/Hispanic	5 (0.6)	49 (43.4)	21 (16.8)	0	118 (40.3)	193 (10.2)
Another Race/Ethnicity	18 (2.3)	10 (8.9)	17 (13.6)	46 (7.8)	12 (4.1)	103 (5.4)
**Current Meth/Amphetamine Use**	43 (5.5)	23 (20.4)	14 (11.2)	311 (52.6)	163 (55.6)	554 (29.1)
**Current Cocaine Use**	460 (59.1)	17 (15.0)	15 (12.0)	434 (73.4)	79 (27.0)	1005 (52.9)
**Current Opioid Use**	474 (60.9)	6 (5.3)	4 (3.2)	412 (69.7)	18 (6.1)	914 (48.1)
**Diagnosed with HIV**	195 (25.0)	25 (22.1)	60 (48.0)	111 (18.8)	160 (54.6)	551 (29.0)
**Depressive Symptoms (≥ Moderate)**	435 (55.8)	31 (27.4)	42 (33.6)	290 (49.1)	189 (64.5)	987 (51.9)
**Gained Housing at Any Follow-Up**	673 (86.4)	107 (94.7)	118 (94.4)	555 (93.9)	249 (85.0)	1702 (89.5)
**Years of Follow-Up**	1.1 (+ 1.2)	0.9 (+ 0.8)	1.0 (+ 1.0)	1.9 (+ 2.2)	1.4 (+ 1.4)	1.4 (+ 1.6)

Among the participants who were housed at baseline, using separate models, current use of amphetamines (hazard ratio [HR]=1.81, 95% confidence interval [CI] 1.47, 2.23), opioids (HR=1.39, 95%CI 1.14, 1.69), and cocaine (HR=1.68, 95%CI 1.39, 2.02) were associated with a significantly higher risk of becoming unhoused (Table 3). There was some evidence of heterogeneity in cohort specific estimates for amphetamines (I^2^=41%), cocaine (I^2^=54%), and opioids (I^2^=35%). Looking at individual cohort point estimates for cocaine (only I^2^>50%), the heterogeneity for cocaine was likely driven by the lower point estimates observed in the RADAR and HYM/TRUTH cohorts. Any MCO substance was also associated with faster loss of housing (HR=1.76, 95% CI:1.56, 1.99) and this association was consistent across the cohorts (Table 4).

**Table 3:** Association between substance use and transitions from housed to unhoused (model set 1) and unhoused to housed (model set 2).

**Substance**	**ALIVE** **HR** **(95% CI)**	**HYM/TRUTH** **HR** **(95% CI)**	**RADAR** **HR** **(95% CI)**	**ACCESS/ARYS/VIDUS** **HR** **(95% CI)**	**mSTUDY** **HR** **(95% CI)**	**I**^**2**^	**Overall** **HR** **(95% CI)**
Model Set 1	First Housed Instance -> Unhoused						
**Amphetamines**	1.94 (1.25, 3.01)	2.05 (1.31, 3.22)	1.51 (0.87, 2.62)	1.49 (1.20, 1.83)	2.27 (1.70, 3.02)	41%	1.81 (1.47, 2.23)
***Weights***	*15.4*	*14.9*	*10.9*	*33.3*	*25.4*		
**Cocaine**	1.78 (1.50, 2.12)	1.19 (0.74, 1.92)	1.00 (0.57, 1.74)	1.29 (1.05, 1.60)	1.34 (0.97, 1.86)	54%	1.39 (1.14, 1.69)
***Weights***	*31.0*	*12.0*	*9.6*	*28.0*	*19.4*		
**Opioids**	1.76 (1.48, 2.11)	2.81 (1.38, 5.69)	2.02 (0.95, 4.28)	1.41 (1.17, 1.70)	1.76 (1.04, 2.98)	35%	1.68 (1.39, 2.02)
***Weights***	*39.7*	*6.2*	*5.5*	*38.2*	*10.4*		
Model Set 2	First Unhoused Instance -> Housed						
**Amphetamines**	1.01 (0.69, 1.48)	0.70 (0.44, 1.11)	0.79 (0.47, 1.31)	0.82 (0.69, 1.00)	0.71 (0.54, 0.93)	0%	0.80 (0.70, 0.91)
***Weights***	*12.3*	*8.6*	*6.9*	*48.4*	*23.9*		
**Cocaine**	0.83 (0.72, 0.95)	0.78 (0.50, 1.23)	0.73 (0.37, 1.45)	0.91 (0.76, 1.10)	0.73 (0.56, 0.97)	0%	0.83 (0.75, 0.92)
***Weights***	*50.9*	*5.0*	*2.2*	*28.2*	*13.7*		
**Opioids**	0.89 (0.77, 1.03)	0.68 (0.29, 1.60)	0.48 (0.16, 1.45)	0.83 (0.68, 1.00)	0.70 (0.43, 1.13)	0%	0.85 (0.76, 0.95)
***Weights***	*56.3*	*1.7*	*1.0*	*35.5*	*5.5*		

All models adjusted for age, sex, race/ethnicity and year of study enrollment

Illicit opioids, methamphetamine/amphetamine, and cocaine/crack are the precise substance use categories, simplified for the table

**Table 4:** Association between substance use and transitions from housed to unhoused (model set 1) and unhoused to housed (model set 2).

**Substance**	**ALIVE** **HR** **(95% CI)**	**HYM/TRUTH** **HR** **(95% CI)**	**RADAR** **HR** **(95% CI)**	**ACCESS/ARYS/VIDUS** **HR** **(95% CI)**	**mSTUDY** **HR** **(95% CI)**	**I**^**2**^	**Overall** **HR** **(95% CI)**
Model Set 1	First Housed Instance -> Unhoused						
**MCO use**	1.86 (1.56, 2.23)	1.42 (0.94, 2.14)	1.43 (0.94, 2.17)	1.83 (1.38, 2.43)	1.79 (1.36, 2.35)	0%	1.76 (1.56, 1.99)
***Weights***	*45.7*	*8.7*	*8.3*	*18.0*	*19.4*		
Model Set 2	First Unhoused Instance -> Housed						
**MCO use**	0.88 (0.76, 1.02)	0.75 (0.51, 1.09)	0.73 (0.47, 1.14)	0.89 (0.63, 1.27)	0.65 (0.50, 0.83)	34%	0.79 (0.68, 0.92)
***Weights***	*38.8*	*13.1*	*10.1*	*14.4*	*23.6*		

All models adjusted for age, sex, race/ethnicity, year of study enrollment, and moderate or more severe depressive symptoms

MCO use consists of Illicit opioids, methamphetamine/amphetamine, and/or cocaine/crack

Among the participants who were unhoused at baseline, amphetamines (HR=0.80, 95% CI 0.70, 0.91) opioids (HR = 0.83, 95%CI 0.75, 0.92) and cocaine (HR=0.85, 95% CI 0.76, 0.95) were associated with regaining housing at a lower rate (Table 3). Unlike the housed participants, there was no evidence of heterogeneity (I^2^=0%) between any of the cohort specific estimates. Any MCO substance was also associated with slower regaining of housing (HR=0.79, 95% CI: 0.68, 0.92) and this association was consistent across the cohorts (although it only reached individual cohort statistical significance for the mSTUDY cohort).

All estimates were consistent when adjusted for depressive symptoms (Supplemental table 1) and when adjusted for HIV status (Supplemental table 2), although cocaine and amphetamines showed weaker associations with time to loss of housing when depressive symptoms were accounted for. Substance use by frequency (less than weekly versus weekly or more frequently) showed much faster time to becoming unhoused for the more frequent substance users. Methamphetamine use was associated with loss of housing HR= 1.52 (95% CI: 1.03, 2.24) for less than weekly compared to no use and even more strongly for more frequently than weekly use as compared to no use HR= 2.06 (95% CI: 1.22, 3.48). Cocaine use was not associated with loss of housing HR= 1.09 (95% CI: 0.89, 1.33) for less than weekly compared to no use and was associated with loss of housing HR= 1.60 (95% CI: 1.22, 3.48) for more frequently than weekly use as compared to no use. Opioid use was not associated with loss of housing HR= 1.20 (95% CI: 0.95, 1.52) for less than weekly compared to no use but was associated with a loss of housing then used more frequently than weekly use as compared to no use HR= 1.55 (95% CI: 1.26, 1.90). However, frequency of substance use was less important for estimating unhoused participants’ time to regaining housing (Supplemental Table 3), with estimates being broadly similar to results that did not account for frequency of use. Missing data rates for the non-outcome variables were generally low, except for some missing methamphetamine data in the ALIVE cohort (Supplemental Table 4).

## 4 Discussion

We observed that current opioid, amphetamine, and cocaine use are associated with both the loss of existing housing and with challenges in becoming rehoused once a participant has lost housing. This is important as housing instability may lead to increased substance use (Austin et al., 2021), further impeding efforts for unhoused participants to become housed, and highlights the need for improved harm reduction services and health care outreach to these vulnerable populations (Kalicum et al., 2025, Vilsaint et al., 2025).

The existing literature has diverse estimates for global substance use (all substances) in different target populations. One possible reason for heterogeneity is different compositions of substance use in different times and places, which makes substance specific estimates important. For example, a meta-analysis showed a larger effect size than we did for the association between substance use and becoming unhoused using an odds ratio (OR) to estimate measures of association (OR=2.3, 95% CI: 1.5–3.5) (Nilsson et al., 2019). However, the study also noted an I^2^ of 99.7%, suggesting extreme levels of heterogeneity with the inclusion of a single definition of substance use (Nilsson et al., 2019). Some studies like McVicar et al. (2015) found no association between illicit drug use and incident loss of housing. On the other hand, the same meta-analysis found very similar association of substance use and regaining housing (OR=0.8, 95% CI: 0.7–0.9), with far lower heterogeneity (I^2^=17%) (Nilsson et al., 2019), which is consistent with our pattern of results. Another meta-analysis that looked at substance use as a type of mental health disorder demonstrated very high levels of heterogeneity (Barry et al., 2024), which further makes it important to consider type of substance to ensure that heterogeneity was not driven by differences in substances used. Prior work in the ALIVE cohort has shown similar results with injection drug use (Shah et al., 2006, Linton et al., 2013) with very similar results to our overall estimates of the association between substance use and time to loss of housing. Our pooled results where we combine the substances into an “any use” exposure, as shown in Table 4, are broadly aligned with the existing literature.

There was some heterogeneity observed in the estimates for the time to loss of housing for both cocaine and amphetamines. It is notable that the cohorts that do not show this association for cocaine consist of younger participants (mean age 21 and 23, versus 50 for ALIVE, 32 for mSTUDY, and 39 for the three Vancouver cohorts), which might be suggestive of an age effect when seeing estimate heterogeneity or some other factor we were unable to capture. The lower cocaine estimates from the younger cohorts (RADAR, HYM/TRUTH, and potentially even mSTUDY) might suggest attenuation among younger participants and it is notable that this estimate was the only estimate that had an I^2^ above 50% (although others were close to 50%). This could potentially suggest some resilience to cocaine outcomes in younger persons with a substance use disorder or issues associated with cocaine use that take time to emerge such as accelerated aging (Beheshti, 2023, Schinz et al., 2023, Rothmann et al., 2024), which might well account for the unexpected null association in these cohorts. Equally, it could be due to some other factor, that we were not able to capture, that differentially confounded persons who use cocaine as compared to persons who use opioids and methamphetamines in some of the cohorts.

A key value of the estimates in this study is that they generate plausible effect sizes for interventions targeting substance use. Some substances, such as opioids, have well established treatments that have known efficacy (Macnaughton et al., 2018). Other substances have less clear and high quality evidence for interventions (Khalili et al., 2025) meaning that direct treatment of the substance use disorder may be more challenging. It is notable that the substance we considered with the least evidence for treatment options (methamphetamine) is the one with the fastest time to loss of housing and the slowest time to regain housing in our study. This observation suggest that approaches like Housing First (O’Campo et al., 2022) may be helpful as they may bypass the lack of pharmacological or behavioral therapy options for these substances. Given that estimates for time to regain housing are consistent across cohorts, this may make these sorts of policies attractive as targets for intervention studies in the currently unhoused.

In particular, policies like Housing First (O’Campo et al., 2022) may be able to make an important harm reduction contribution in populations who use substances lacking clear evidenced-based therapies for substance use disorder treatment. It can do this assisting people who use these substances in finding access to stable housing as a first step towards successfully addressing substance use disorders. It is notable that the ACCESS, ARYS, and VIDUS cohorts, situated in Vancouver, generally show slightly less magnitude of association between substance use and time to rehousing than most of the cohorts in the United States (the exception being ALIVE). It is possible that this represents differences in Canada in terms of policy and/or environment, which has different housing initiatives than the United States (Baines et al., 2025, Kerman et al., 2025, Redline et al., 2025).

It is also the case that improved access to substance use treatments might also be worth testing as a policy intervention to improve the trajectory of acquiring stable housing among people who use drugs (Ivsins et al., 2022, Vilsaint et al., 2025). However, this needs to be considered as a part of a holistic approach to housing and substance use. Some studies have found evidence that focusing on substance use treatment before housing shows inferior outcomes to a Housing First approach (Tsemberis et al., 2004, Padgett et al., 2011, Peng et al., 2020) and this should continue to be evaluated. One goal of this area of research is to continue to understand the complex interplay between substance use and housing so that we can better craft policy and health care interventions to support some of the most vulnerable populations. It is likely that some combination of housing and substance use care and support will be critical to success in these endeavors, and not just one or the other.

That said, this heterogeneity could also limit the transportability of this estimate to other populations. We don’t have the statistical power or the breadth of covariates to rule out the presence of effect measure modifiers (Dahabreh et al., 2020) and it is possible that these estimates may not apply to the entire US and Canadian population of people who use substances. For these reasons, while these estimates are more generalizable than a single cohort estimate, it may be best to not interpret these associations too strongly in a causal framework (Barker et al., 2022). Instead, it is wiser to consider them as associations that require proper testing via implementation science approaches (Macnaughton et al., 2018) before they are considered to be transportable to the full target population. Such effect measure modifiers are plausible as a major contribution of this study is to show that associations with becoming unhoused are modified by type of substance (Table 3). It is not implausible that other modifiers may well be present in these associations given the complexity of these associations.

This study has limitations. Our substance use definitions were limited by the questions asked in the cohort studies. We were limited in our ability to explore the role of mental health in these associations and were able to consider just depressive symptoms. However, depressive symptoms showed minimal influence on the size of estimates in sensitivity analysis as did HIV status. We were also limited in our ability to explore the role of other social and structural factors (e.g., criminal justice system involvement, use of substance use treatment) in these associations. These estimates are based on observational data collected by self-report: there will always be some measurement error in these estimates and the possibility of unmeasured confounders is always a concern. Our estimates are restricted to urban cities and may not extend to rural populations (Jenkins et al., 2022). We only looked at substance use and housing but not how the experience of being unhoused can drive substance use(McVicar et al., 2015), as other authors have found the challenges of being unhoused may be a factor in the use of substances. There are some limitations to the statistical approach including the six month window for both exposure and outcome being measured only at study visits, calendar time periods covered by the studies varying and there being a risk of some residual confounding. Further, the use of single substance models (because of collinearity) do not support direct comparisons between substances, the reason for loss to follow is not known (and might include death), and finally, despite harmonization efforts (Gorbach et al., 2021), there remain some differences in definitions across cohorts in how the substance use questions are asked. Our confounder adjustment strategy did not consider time-dependent confounders, although our expert-based DAG (Shrier and Platt, 2008) did not suggest the presence of any of these factors. Finally, there was some heterogeneity in the meta-analytic pooling of results for some of the substance use and time to becoming unhoused associations (e.g., cocaine) that could use additional investigation in other cohorts or contexts.

This study also has some notable strengths. It looks at an important vulnerable population across a range of geographic settings. It uses validated instruments to assess substance use (Gorbach et al., 2021) and depressive symptoms(Kroenke et al., 2001; Kim et al., 2017). It includes both people with and without HIV, making it applicable to multiple important populations among people who use drugs. Finally, these are long established cohorts studying substance use and allow for substantial follow-up time to estimate time to change in housing status.

In conclusion, there is an opportunity to further improve housing outcomes among vulnerable populations of people who use drugs in the United States and Canada. Further studies could help understand the mechanism between heterogeneity among substance use and those who are at risk of losing housing in different contexts, as well as better understand the bidirectional relationship between housing and substance use by examining associations in the opposite direction as many have argued that loss of housing is a cause of substance use among the unhoused (McVicar et al., 2015). Looking at substance by type led to some intriguing possibilities, including that cocaine use may not show immediate adverse associations on this outcome among younger populations. There is also a key opportunity to better integrate harm reduction policies to improve outcomes among participants. Further, these results give reasonable bounds on the potential for interventions to speed up the rate at unhoused people who use drugs can regain housing by mitigating the association of substance use on housing availability.

## CRediT authorship contribution statement

**Michele Kipke:** Writing – review & editing, Funding acquisition, Conceptualization. **Madison Shea Smith:** Writing – review & editing, Funding acquisition, Conceptualization. **Patrick Janulis:** Writing – review & editing. **M-J Milloy:** Writing – review & editing, Funding acquisition, Conceptualization. **Ryan P Kyle:** Writing – review & editing, Investigation, Conceptualization. **Michael E Newcomb:** Writing – review & editing, Funding acquisition, Conceptualization. **Bridget M Whitney:** Writing – review & editing, Supervision, Data curation, Conceptualization. **Lydia N Drumright:** Writing – review & editing, Supervision, Project administration, Funding acquisition, Formal analysis, Data curation, Conceptualization. **Carolyn A Fahey:** Writing – review & editing, Methodology, Investigation. **Pamina M. Gorbach:** Writing – review & editing, Funding acquisition, Conceptualization. **Brenton Swenson:** Writing – review & editing, Formal analysis, Data curation. **Marjan Javanbakht:** Writing – review & editing, Investigation, Conceptualization. **Raul Mandler:** Writing – review & editing, Supervision, Conceptualization. **Gregory Kirk:** Writing – review & editing, Funding acquisition, Conceptualization. **Brian Mustanski:** Writing – review & editing, Funding acquisition, Conceptualization. **Delaney Joseph A C:** Writing – original draft, Supervision, Methodology, Funding acquisition, Conceptualization. **Geetanjali Chander:** Writing – review & editing, Supervision, Investigation, Funding acquisition, Conceptualization. **Becky L Genberg:** Writing – review & editing, Investigation, Conceptualization. **Kanna Hayashi:** Writing – review & editing, Investigation, Funding acquisition, Conceptualization. **Shruti Metha:** Writing – review & editing, Funding acquisition, Conceptualization. **Heidi M Crane:** Writing – review & editing, Supervision, Resources, Investigation, Funding acquisition, Conceptualization. **Kora DeBeck:** Writing – review & editing, Funding acquisition, Conceptualization.

## Funding

Funding for this study was provided from 10.13039/100000026NIDA via the HIV SUCCESS Coordinating Center (U24DA058307), ACCESS, ARYS, and VIDUS (U01DA021525, U01DA038886), ALIVE (U01DA036297), HYM/TRUTH (U01DA036926), mSTUDY (U01DA036267), and RADAR (U01DA036939), and KH holds the St. Paul’s Hospital Chair in Substance Use Research and is supported in part by the St. Paul’s Foundation.

## Declaration of Competing Interest

The authors declare the following financial interests/personal relationships which may be considered as potential competing interests: Joseph Delaney reports financial support was provided by National Institute on Drug Abuse. If there are other authors, they declare that they have no known competing financial interests or personal relationships that could have appeared to influence the work reported in this paper.
