# Supplementary material for: Illicit opioids, methamphetamine/amphetamine, and cocaine use are associated with loss of housing and delay with becoming rehoused across seven diverse cohorts with and without HIV in the United States and Canada
Source: Drug Alcohol Depend Rep. 2026 Oct 5;21:100483. doi: 10.1016/j.dadr.2026.100483 (PMC13676702; doi:10.1016/j.dadr.2026.100483)
Supplement: Supplementary file 1 [file mmc1.docx]

**Supplemental Table 1: Association between substance use and transitions from housed to unhoused (model set 1) and unhoused to housed (model set 2) additionally adjusted for moderate or more severe depressive symptoms**

| **Substance** | **ALIVE**  **HR**  **(95% CI)** | **HYM+TRUTH**  **HR**  **(95% CI)** | **RADAR**  **HR**  **(95% CI)** | **ACCESS/ARYS/VIDUS**  **HR**  **(95% CI)** | **mSTUDY**  **HR**  **(95% CI)** | **I^2^** | **Overall**  **HR**  **(95% CI)** |
| --- | --- | --- | --- | --- | --- | --- | --- |
| **Model Set 1** | **First Housed Instance -> Unhoused** | | | | | | |
| **Amphetamines** | 1.87  (1.21, 2.87) | 2.01  (1.29, 3.14) | 1.36  (0.78, 2.38) | 1.48  (1.20, 1.83) | 2.01  (1.49, 2.70) | 20% | 1.71  (1.44, 2.03) |
| ***Weights*** | *13.8* | *13.0* | *8.7* | *39.4* | *25.1* |  |  |
| **Cocaine** | 1.71  (1.43, 2.04) | 1.15  (0.72, 1.86) | 0.93  (0.54, 1.62) | 1.29  (1.05, 1.59) | 1.28  (0.93, 1.76) | 51% | 1.35  (1.11, 1.63) |
| ***Weights*** | *31.3* | *11.6* | *9.3* | *28.2* | *19.6* |  |  |
| **Opioids** | 1.68  (1.40, 2.01) | 2.78  (1.37, 5.64) | 1.80  (0.85, 3.82) | 1.41  (1.17, 1.70) | 1.56  (0.92, 2.64) | 15% | 1.59  (1.37, 1.85) |
| ***Weights*** | *43.3* | *4.3* | *3.8* | *41.1* | *7.5* |  |  |
| **Model Set 2** | **First Unhoused Instance -> Housed** | | | | | | |
| **Amphetamines** | 1.03  (0.71, 1.50) | 0.71  (0.46, 1.10) | 0.81  (0.48, 1.36) | 0.83  (0.69, 1.01) | 0.75  (0.57, 0.98) | 0% | 0.82  (0.72, 0.94) |
| ***Weights*** | *12.2* | *9.0* | *6.5* | *48.2* | *24.1* |  |  |
| **Cocaine** | 0.84  (0.73, 0.97) | 0.75  (0.47, 1.20) | 0.75  (0.39, 1.45) | 0.94  (0.78, 1.13) | 0.79  (0.60, 1.04) | 0% | 0.86  (0.77, 0.95) |
| ***Weights*** | *49.5* | *4.6* | *2.4* | *29.9* | *13.5* |  |  |
| **Opioids** | 0.91  (0.78, 1.06) | 0.68  (0.30, 1.56) | 0.50  (0.17, 1.44) | 0.84  (0.70, 1.01) | 0.75  (0.46, 1.21) | 0% | 0.87  (0.77, 0.97) |
| ***Weights*** | *55.8* | *1.8* | *1.1* | *35.8* | *5.4* |  |  |

***All models adjusted for age, sex, race/ethnicity, year of study enrollment, and moderate or more severe depressive symptoms***

Illicit opioids, methamphetamine/amphetamine, and cocaine/crack are the precise substance use categories, simplified for the table

**Supplemental Table 2: Association between substance use and transitions from housed to unhoused (model set 1) and unhoused to housed (model set 2) additionally adjusted for HIV Status**

| **Substance** | **ALIVE**  **HR**  **(95% CI)** | **HYM+TRUTH**  **HR**  **(95% CI)** | **RADAR**  **HR**  **(95% CI)** | **ACCESS/ARYS/VIDUS**  **HR**  **(95% CI)** | **mSTUDY**  **HR**  **(95% CI)** | **I^2^** | **Overall**  **HR**  **(95% CI)** |
| --- | --- | --- | --- | --- | --- | --- | --- |
| **Model Set 1** | **First Housed Instance -> Unhoused** | | | | | | |
| **Amphetamines** | 1.94  (1.25, 3.01) | 1.89  (1.17, 3.05) | 1.41  (0.81, 2.46) | 1.50  (1.21, 1.85) | 2.32  (1.72, 3.12) | 41% | 1.79  (1.45, 2.21) |
| ***Weights*** | *15.8* | *14.0* | *11.2* | *33.7* | *25.3* |  |  |
| **Cocaine** | 1.78  (1.50, 2.12) | 1.21  (0.75, 1.95) | 0.96  (0.54, 1.70) | 1.29  (1.05, 1.59) | 1.36  (0.98, 1.89) | 54% | 1.39  (1.14, 1.70) |
| ***Weights*** | *31.0* | *12.1* | *9.3* | *28.2* | *19.4* |  |  |
| **Opioids** | 1.76  (1.47, 2.11) | 2.73  (1.36, 5.48) | 1.92  (0.87, 4.20) | 1.38  (1.14, 1.67) | 1.76  (1.04, 2.97) | 39% | 1.67  (1.37, 2.02) |
| ***Weights*** | *39.0* | *6.9* | *5.5* | *37.6* | *11.0* |  |  |
| **Model Set 2** | **First Unhoused Instance -> Housed** | | | | | | |
| **Amphetamines** | 1.01  (0.69, 1.48) | 0.69  (0.43, 1.10) | 0.80  (0.48, 1.33) | 0.82  (0.68, 0.99) | 0.69  (0.52, 0.91) | 0% | 0.80  (0.69, 0.91) |
| ***Weights*** | *12.5* | *8.3* | *7.0* | *49.4* | *22.8* |  |  |
| **Cocaine** | 0.83  (0.72, 0.95) | 0.78  (0.50, 1.23) | 0.71  (0.36, 1.42) | 0.91  ().75, 1.10) | 0.76  (0.58, 0.98) | 0% | 0.83  (0.75, 0.92) |
| ***Weights*** | *50.2* | *4.9* | *2.1* | *28.3* | *14.5* |  |  |
| **Opioids** | 0.89  (0.77, 1.04) | 0.67  (0.29, 1.57) | 0.47  (0.15, 1.45) | 0.83  (0.68, 1.00) | 0.71  (0.44, 1.15) | 0% | 0.85  (0.76, 0.95) |
| ***Weights*** | *56.4* | *1.8* | *1.0* | *35.3* | *5.5* |  |  |

***All models adjusted for age, sex, race/ethnicity and year of study enrollment***

* Illicit opioids, methamphetamine/amphetamine, and cocaine/crack are the precise substance use categories, simplified for the table

**Supplemental Table 3: Association between frequency of substance use and transitions from housed to unhoused (model set 1) and unhoused to housed (model set 2)**

| **Substance** | **HYM+TRUTH**  **HR (95% CI)** | **RADAR**  **HR (95% CI)** | **ACCESS/ARYS/VIDUS**  **HR (95% CI)** | **mSTUDY**  **HR (95% CI)** | **I^2^** | **Overall**  **HR (95% CI)** |
| --- | --- | --- | --- | --- | --- | --- |
| **Model Set 1** | **First Housed Instance -> Unhoused** | | | | | |
| **Amphetamines** |  |  |  |  |  |  |
| **Weekly vs none** | 1.20 (0.58, 2.45) | 0.90 (0.39, 2.03) | 1.44 (1.08, 1.91) | 2.32 (1.68, 3.20) | 64% | 1.52 (1.03, 2.24) |
| ***(N <weekly use)ⱡ*** | *(N=50)* | *(N=50)* | *(N=102)* | *(N=102)* |  | *(N=312)* |
| ***Weights*** | *17.4* | *14.6* | *34.8* | *33.2* |  |  |
| **≥Weekly vs none** | 4.37 (2.60, 7.32) | 1.17 (0.56, 2.45) | 1.52 (1.19, 1.94) | 2.20 (1.52, 3.20) | 84% | 2.06 (1.22, 3.48) |
| ***(N ≥weekly use)ⱡ*** | *(N=25)* | *(N=58)* | *(N=177)* | *(N=76)* |  | *(N=337)* |
| ***Weights*** | *24.1* | *19.4* | *29.4* | *27.2* |  |  |
| **Cocaine** |  |  |  |  |  |  |
| **>Weekly vs none** | 0.91 (0.50, 1.64) | 0.64 (0.26, 1.57) | 1.07 (0.82, 1.40) | 1.30 (0.91, 1.85) | 0% | 1.09 (0.89, 1.33) |
| ***(N <weekly use)ⱡ*** | *(N=63)* | *(N=71)* | *(N=138)* | *(N=81)* |  | *(N=360)* |
| ***Weights*** | *11.0* | *4.9* | *52.8* | *31.3* |  |  |
| **≥Weekly vs none** | 3.13 (1.43, 6.82) | 1.65 (0.83, 3.28) | 1.46 (1.16, 1.85) | 1.55 (0.77, 3.13) | 8% | 1.60 (1.26, 2.02) |
| ***(N ≥weekly use)ⱡ*** | *(N=18)* | *(N=34)* | *(N=327)* | *(N=19)* |  | *(N=452)* |
| ***Weights*** | *8.8* | *11.1* | *69.3* | *10.8* |  |  |
| **Opioids** |  |  |  |  |  |  |
| **>Weekly vs none** | 1.47 (0.46, 4.68) | 1.65 (0.80, 3.42) | 1.09 (0.84, 1.42) | 1.46 (0.79, 2.70) | 3% | 1.20 (0.95, 1.52) |
| ***(N <weekly use)ⱡ*** | *(N=24)* | *(N=51)* | *(N=138)* | *(N=15)* |  | *(N=234)* |
| ***Weights*** | *4.2* | *10.3* | *71.1* | *14.4* |  |  |
| **≥Weekly vs none** | NA* | 1.55 (0.53, 4.57) | 1.55 (1.25, 1.91) | NA* | 0% | 1.55 (1.26, 1.90) |
| ***(N ≥weekly use)ⱡ*** | *(N=5)* | *(N=19)* | *(N=296)* | *(N=5)* |  | *(n=315)* |
| ***Weights*** |  | *3.7* | *96.3* |  |  |  |
| **Model Set 2** | **First Unhoused Instance -> Housed** | | | | | |
| **Amphetamines** |  |  |  |  |  |  |
| **>Weekly vs none** | 0.55 (0.25, 1.24) | 0.98 (0.62, 1.55) | 1.07 (0.82, 1.39) | 0.83 (0.60, 1.15) | 5% | 0.94 (0.78, 1.13) |
| ***(N <weekly use)ⴕ*** | *(N=7)* | *(N=8)* | *(N=100)* | *(N=67)* |  | *(N=193)* |
| ***Weights*** | *5.4* | *16.6* | *46.7* | *31.4* |  |  |
| **≥Weekly vs none** | 0.84 (0.52, 1.34) | 0.72 (0.37, 1.41) | 0.69 (0.55, 0.86) | 0.62 (0.45, 0.85) | 0% | 0.69 (0.58, 0.81) |
| ***(N ≥weekly use)ⴕ*** | *(N=12)* | *(N=10)* | *(N=202)* | *(N=96)* |  | *(N=327)* |
| ***Weights*** | *12.2* | *5.9* | *56.0* | *25.9* |  |  |
| **Cocaine** |  |  |  |  |  |  |
| **>Weekly vs none** | 0.60 (0.35, 1.05) | 0.62 (0.26, 1.44) | 0.87 (0.69, 1.11) | 0.79 (0.60, 1.04) | 0% | 0.80 (0.68, 0.95) |
| ***(N <weekly use)ⴕ*** | *(N=6)* | *(N=11)* | *(N=140)* | *(N=69)* |  | *(N=235)* |
| ***Weights*** | *9.5* | *4.0* | *49.8* | *36.8* |  |  |
| **≥Weekly vs none** | NA* | NA* | 0.94 (0.75, 1.17) | 0.48 (0.24, 0.99) | 67% | 0.74 (0.39, 1.38) |
| ***(N ≥weekly use)ⴕ*** | *(N=4)* | *(N=4)* | *(N=287)* | *(N=10)* |  | *(N=297)* |
| ***Weights*** |  |  | *63.6* | *36.4* |  |  |
| **Opioids** |  |  |  |  |  |  |
| **>Weekly vs none** | NA* | 0.87 (0.45, 1.67) | 0.97 (0.76, 1.24) | 0.82 (0.47, 1.44) | 0% | 0.94 (0.76, 1.16) |
| ***(N <weekly use)ⴕ*** | *(N=3)* | *(N=9)* | *(N=112)* | *(N=14)* |  | *(N=125)* |
| ***Weights*** |  | *10.3* | *75.9* | *13.9* |  |  |
| **≥Weekly vs none** | NA* | NA* | 0.77 (0.63, 0.95) | NA* |  | 0.77 (0.63, 0.95) |
| ***(N ≥weekly use)ⴕ*** | *(N=1)* | *(N=1)* | *(N=290)* | *(N=4)* |  | *(N=290)* |
| ***Weights*** |  |  | *100* |  |  |  |

***All models adjusted for age, sex, race/ethnicity and year of study enrollment***

Illicit opioids, methamphetamine/amphetamine, and cocaine/crack are the precise substance use categories, simplified for the table

ⱡN at first housed visit	***ⴕ***N at first unhoused visit	*Not applicable, N too small to reliably estimate

**Supplemental Table 4:** Missing data by variable for the non-outcome measures

| **Variables in Housed to Unhoused analysis** | **Number Missing** |
| --- | --- |
| Methamphetamine use | 1,180 |
| Cocaine | 15 |
| Opioids | 11 |
| Age | 0 |
| Sex | 0 |
| Race/ethnicity | 0 |
| Enrollment Year | 0 |
| Depression | 14 |
| HIV Status | 0 |
| **Variables in Unhoused to Housed analysis** | **Number Missing** |
| Methamphetamine | 345 |
| Cocaine | 5 |
| Opioids | 4 |
| Age | 0 |
| Sex | 0 |
| Race/ethnicity | 0 |
| Enrollment Year | 0 |
| Depression | 4 |
| HIV Status | 0 |
